# Dichotomic Pattern Mining Integrated With Constraint Reasoning for Digital Behavior Analysis

**DOI:** 10.3389/frai.2022.868085

**Published:** 2022-07-12

**Authors:** Sohom Ghosh, Shefali Yadav, Xin Wang, Bibhash Chakrabarty, Serdar Kadıoğlu

**Affiliations:** ^1^AI Center of Excellence, Fidelity Investments, Boston, MA, United States; ^2^Department of Computer Science, Brown University, Providence, RI, United States

**Keywords:** dichotomic pattern mining, sequential pattern mining, semi-structured clickstream datasets, digital behavior analysis, intent prediction, intrusion detection, constraint-based sequential pattern mining, knowledge extraction and representation

## Abstract

Sequential pattern mining remains a challenging task due to the large number of redundant candidate patterns and the exponential search space. In addition, further analysis is still required to map extracted patterns to different outcomes. In this paper, we introduce a pattern mining framework that operates on semi-structured datasets and exploits the dichotomy between outcomes. Our approach takes advantage of constraint reasoning to find sequential patterns that occur frequently and exhibit desired properties. This allows the creation of novel pattern embeddings that are useful for knowledge extraction and predictive modeling. Based on dichotomic pattern mining, we present two real-world applications for customer intent prediction and intrusion detection. Overall, our approach plays an integrator role between semi-structured sequential data and machine learning models, improves the performance of the downstream task, and retains interpretability.

## 1. Introduction

Sequential Pattern Mining (SPM) is highly relevant in various practical applications including the analysis of medical treatment history (Bou Rjeily et al., 2019), customer purchases (Requena et al., 2020), segmentation (Kuruba Manjunath and Kashef, 2021), call patterns, and digital clickstream (Agrawal and Srikant, 1995; Srikant and Agrawal, 1996). A recent survey that covers SPM fundamentals and applications can be found in Gan et al. (2019). In SPM, we are given a set of sequences that is referred to as *sequence database*. As shown in the example in Table 1, each sequence is an ordered set of *items*. Each item might be associated with a set of *attributes* to capture item properties, e.g., price, timestamp. A *pattern* is a subsequence that occurs in at least one sequence in the database maintaining the original ordering of items. The number of sequences that contain a pattern defines the *frequency*. Given a sequence database, SPM is aimed at finding patterns that occur more than a certain frequency threshold.

**Table 1:** Example sequence database with three sequences and two attributes, price and timestamp.

**Sequence database 〈(item, price, timestamp)〉**
〈(A, 5, 1), (A, 5, 1), (B, 3, 2), (A, 8, 3), (D, 2, 3)〉
〈(C, 1, 3), (B, 3, 8), (A, 3, 9)〉
〈(C, 4, 2), (A, 5, 5), (C, 2, 5), (D, 1, 7)〉

In practice, finding the entire set of frequent patterns in a sequence database is not the ultimate goal. The number of patterns is typically too large and may not provide significant insights. It is thus important to search for patterns that are not only frequent but also capture specific properties of the application at hand. This has motivated research in Constraint-based SPM (CSPM) (Pei et al., 2007; Chen et al., 2008). The goal of CSPM is to incorporate constraint reasoning into sequential pattern mining to find smaller subsets of interesting patterns.

As an example, let us consider online retail clickstream analysis. We might not be interested in all frequent browsing patterns. For instance, the pattern 〈*login, logout*〉 is likely to be frequent but offers little value. Instead, we seek recurring clickstream patterns with unique properties, e.g., frequent patterns from sessions where users spend at least a minimum amount of time on a particular set of items with a specific price range. Such constraints help reduce the search space for the mining task and help discover patterns that are more effective in knowledge discovery than arbitrarily frequent clickstreams.

In this paper, our algorithmic focus is on embedding constraint-based sequential patterns in a framework that exploits the dichotomy of positive vs. negative outcomes in user cohorts. We refer to this framework as Dichotomic Pattern Mining (DPM) and present it in detail in Section 4. DPM offers several benefits over SPM and CSPM as discussed in Section 4.1. Our practical focus is on industry-relevant applications of digital behavior analysis. Specifically, we consider two scenarios of predicting the intent of users. The first application is a shopper vs. non-shopper identification in e-commerce applications. The second application is the detection of hostile vs non-hostile users in intrusion detection for security applications. The experimental results given in Section 7 demonstrate that our DPM framework yields significant improvements on such prediction tasks when compared to traditional approaches such as SPM and CSPM as well as modern machine learning approaches such as LSTMs (Hochreiter and Schmidhuber, 1997).

More broadly, the analysis of digital behavior is an integral part of designing online experiences that are geared toward user needs. Customer intent prediction and intrusion detection are particularly important knowledge discovery tasks. Successful applications enabled by DPM hold the potential to boost performance and security in various domains including recommendation systems in e-commerce, virtual agents in retail, and conversational AI across the enterprise. Our paper contributes to this line of research with the introduction of the DPM framework and applications on digital behavior analysis. Overall, our contributions in this paper can be summarized as follows:

- We introduce the Dichotomic Pattern Mining (DPM) framework that extends our recent work (Wang and Kadioglu, 2022) with further details and experiments.
- We show how to apply DPM in practice as an integration technology between raw data and machine learning models for downstream prediction tasks.
- We demonstrate two successful applications of DPM for digital behavior analysis on shopping intent prediction and intrusion detection. We choose these two applications because (i) we seek real-world dataset for industrially-relevant applications, and (ii) we seek different behavior characteristics. The intrusion detection targets a rare-event identification while the e-commerce dataset is relatively more balanced in terms of shopping behavior.

In the remainder, firstly we start with an overview of related topics and we position our work within the literature (Section 2). Secondly we illustrate a pattern mining example (Section 3) to make the ideas of SPM and CSPM more concrete. We then present the details of our dichotomic pattern mining framework and highlight its benefits(Section 4). Next, we share a system architecture (Section 5) that shows DPM as an integration technology. We focus on digital behavior analysis (Section 6) and demonstrate two real-world applications (Section 7) where DPM serves as an integrator between raw data and machine learning models in downstream tasks for customer intent prediction and intruder detection. Finally, we conclude the paper.

## 2. Related Work

Pattern mining benefits from a wealth of literature and enjoys several practical applications. Historically, sequential pattern mining was introduced in the context of market basket analysis (Agrawal and Srikant, 1995) with several algorithm such as GSP (Srikant and Agrawal, 1996), PrefixSpan (Pei et al., 2001), SPADE (Zaki, 2001), and SPAM (Ayres et al., 2002). Mining the complete set of patterns imposes high computational costs and contains a large number of redundant patterns. Thus, CSPM is proposed to alleviate this problem (Bonchi and Lucchese, 2005; Nijssen and Zimmermann, 2014; Aoga et al., 2017). Constraint Programming and graphical representation of the sequence database have been shown to perform well for CSPM (Guns et al., 2017; Kemmar et al., 2017; Borah and Nath, 2018; Hosseininasab et al., 2019).

Our proposal for Dichotomic Pattern Mining (DPM) is related to the supervised descriptive rule discovery (SDRD) framework (Novak et al., 2009). Specific mining tasks in SDRD include Emerging Pattern Mining (EPM), Subgroup Discovery and Contrast set mining. EPM is a data mining task aimed at the detection of differentiating characteristics between classes (García-Vico et al., 2017; Pellegrina et al., 2019), where the discriminative patterns whose support increases significantly from one class to another are identified. Subgroup discovery identifies subsets of a dataset according to an interesting behavior with respect to certain criteria applied to a property (Atzmueller, 2015). Another closely related approach, contrast set mining, tries to find patterns with the high difference of support across different data groups (Bay and Pazzani, 2001). On one hand, DPM can be seen as a special case of these existing approaches focused on the dichotomy of positive and negative outcomes. On the other hand, DPM offers several unique aspects that contribute to this line of research. First, DPM employs *sequential* pattern mining coupled with constraint-reasoning based on the recent work (Wang and Kadioglu, 2022). This is a unique feature of our application that is not considered before. Second, we envision DPM as an integration technology and provide an end-to-end system architecture that competes with sequence-to-pattern generation and pattern-to-feature generation. Finally, we focus on the analysis of digital behavior and utilize our DPM framework for two real-world applications.

Regarding pattern mining tools to utilize in practice, the Python tech stack lacks readily available libraries. Although a few Python libraries exist for SPM (see, e.g., Dagenais, 2016; Gao, 2019), Seq2Pat is the first CSPM library in Python that supports several anti-monotone and non-monotone constraint types. Unfortunately, other CSPM implementations are either not available in Python, hence missing the opportunity to integrate with ML applications, or limited to a few constraint types, most commonly, gap, maximum span, and regular expressions (Yu and Hayato, 2006; Aoga et al., 2016; Fournier-Viger et al., 2016; Bermingham, 2018). Powered by Seq2Pat, DPM offers a complete end-to-end solution.

## 3. Illustrative Example

Let us present the running example from Table 1 to make the idea behind pattern mining more concrete. We first examine the patterns found by *sequential* mining and then extend it to *constraint-based* mining.

### 3.1. Sequential Pattern Mining

In this example, the database is represented as a list of sequences each with a list of items. There are three sequences over the item set {*A, B, C, D*}. Assume we are searching for patterns that occur in at least two sequences. This is typically referred to as the minimum frequency threshold. When we are not enforcing any constraints, we discover three patterns {[*A, D*], [*B, A*], [*C, A*]} subject to minimum frequency threshold. Notice that each pattern occurs in exactly two sequences satisfying the minimum frequency. More specifically;

- The pattern [*A, D*] is a subsequence of the first and the third sequence.
- The pattern [*B, A*] is a subsequence of the first and the second sequence.
- The pattern [*C, A*] is a subsequence of the second and the third sequence.

### 3.2. Constraint-Based Sequential Pattern Mining

Next, we extend the example with more data to introduce various constraints to enforce desired properties on resulting patterns. As shown in Table 1, we incorporate two attributes; price and timestamp. Conceptually, the idea is to capture frequent patterns in the database from users who have spent at least a minimum amount of time on certain items within specific price ranges. For instance, we can introduce a constraint to restrict the average price of items in a pattern to be between the range [3,4]. The task now becomes constraint-based sequential pattern mining. Initially, we found three patterns {[*A, D*], [*B, A*], [*C, A*]} with a frequency threshold of two. When we introduce the average price [*A, D*] is the only remaining pattern that meets the constraint with the same support. The other patterns do not satisfy the conditions.

Let us examine the details of constraint satisfaction that is entailed for the first pattern. The important observation is that the first sequence in the database exhibits three different subsequences of [*A, D*]. Notice the subsequences exhibit different price averages. In the first sequence, the first and the second occurrence of [*A, D*] has *price*_*average*([5, 2]) = 3.5 while the third occurrence has *price*_*average*([8, 2]) = 5. The first two subsequences are feasible with respect to the price constraint, while the third subsequence is infeasible. One satisfying subsequence suffices for constraint feasibility. Hence the first sequence supports the pattern [*A, D*]. Similarly, the last sequence in the database satisfies the constraint with *price*_*average*([5, 1]) = 3 for the [*A, D*] pattern. Two sequences supporting the pattern for the price average meet the minimum required frequency condition. The other patterns do not satisfy the constraints and frequency conditions.

The technology behind our approach for constraint-based sequential pattern mining is based on Multi-valued Decision Diagrams (MDDs) (Bergman et al., 2016). MDDs are widely used as efficient data structures (Wegener, 2000) and for discrete optimization (Bergman et al., 2016). More recently, MDDs were utilized for CSPM (Hosseininasab et al., 2019) to encode the sequences and associated attributes of the sequence databases. The MDD approach accommodates multiple item attributes, such as price and timestamp in our running example, and various constraint types, such as the average constraint among others. The approach is shown to be competitive with or superior to existing CSPM algorithms in terms of scalability and efficiency (Hosseininasab et al., 2019). We recently released the Seq2Pat^1^ library to make this efficient algorithm accessible to a broad audience with a user-friendly interface (Wang et al., 2022).

The application of Seq2Pat to address CSPM is an integral part of our work. In the next section, we present the details of how to integrate CSPM, *via* Seq2Pat, into the dichotomic pattern mining framework.

## 4. Dichotomic Pattern Mining

We now describe dichotomic pattern mining (DPM^2^) that operates over sequence databases augmented with binary labels denoting positive and negative outcomes. Our experiments in Section 7, consider customer intent prediction and user intrusion behavior as the outcome variables.

Algorithm 1 presents our generic approach for dichotomic pattern mining that encapsulates constraint-based sequential pattern mining as a sub-procedure. The algorithm receives a sequence database, SD, containing *N* sequences {*S*_1_, *S*_2_, …, *S*_*N*_}. Each sequence represents a customer's behaviors in time order, for example, the digital clicks in one session. Sequences are associated with binary labels, Y, indicating the outcome of the *i*-th sequence, $Y_{i}\in Y$ where *i* ∈ {1, 2, ⋯ , *N*}, to be positive (⊤) or negative (⊥), e.g., purchase or non-purchase.

As in our example in Table 1, the items in each sequence are associated with a set of attributes 𝔸 = {A, …, A_|𝔸|_}. There is a set of functions *C*_*type*_(·) imposed on attributes with a certain type of operation. For example, $C_{avg}(A_{price})$≥20 requires a pattern to have minimum average price 20. Similarly, there is a minimum threshold θ as frequency lower bound. Given two sets *A* and *B*, we let *A*\*B* denote removing the elements of *A* that are also in *B* and *A*∩*B* denote the intersection of the two sets.

**Algorithm 1:** Dichotomic pattern mining.

**Input:** *Sequence database* SD = {*S*_1_, *S*_2_, …, *S*_*N*_}
**Input:** *Binary label for sequences* Y, *Y*_*i*_ ∈ Y, *Y*_*i*_ = {⊤, ⊥} and *i*∈{1, 2, ⋯ , *N*}
**Input:** *Minimum frequency threshold for positive and negative sets*, θ_⊤_ and θ_⊥_
**Input:** *Pattern constraints for positive and negative sets*, $C_{type}^{⊤}\left(\cdot \right)$ and $C_{type}^{⊥}\left(\cdot \right)$
**Output:** *Frequent pattern sets* P
**Step 1**. Dichotomic split over the dataset
*Pos*←{*SD*_*i*_∣*Y*_*i*_ = ⊤}
*Neg*←{*SD*_*i*_∣*Y*_*i*_ = ⊥}
**Step 2**. Apply constraint-based frequent pattern mining
$Pos_{frequent}\leftarrow CSPM\left(Pos,C_{type}^{⊤}\left(\cdot \right),\;\theta_{⊤}\right)$
$Neg_{frequent}\leftarrow CSPM\left(Neg,C_{type}^{⊥}\left(\cdot \right),\;\theta_{⊥}\right)$
**Step 3**. Find unique patterns and their interaction
*Pos*_*unique*_←*Pos*_*frequent*_\*Neg*_*frequent*_
*Neg*_*unique*_←*Neg*_*frequent*_\*Pos*_*frequent*_
*PN*_*common*_←*Pos*_*frequent*_∩*Neg*_*frequent*_
**Step 4**. Patterns ready for pattern-to-feature generation
P ← {*Pos*_*unique*_, *Neg*_*unique*_, *PN*_*common*_}
**Step 5**. Return frequent patterns for downstream tasks
return P

Conceptually, our algorithm is straightforward and exploits the dichotomy of outcomes. In Step 1, we split the sequences into positive, *Pos*, and negative *Neg* sets. In Step 2, we apply CSPM on each group separately subject to minimum frequency, θ_⊤_ or θ_⊥_, while satisfying constraints, $C_{type}^{⊤}\left(\cdot \right)$ or $C_{type}^{⊥}\left(\cdot \right)$. Notice that frequent patterns found might overlap. Therefore, we perform a set difference operation in each direction. This allows us to distinguish between recurring patterns that *uniquely* identify the positive and negative populations. The outputs P of the DPM algorithm have three sets of patterns, including the frequent patterns that are unique to positive observations, *Pos*_*unique*_, the frequent patterns that are unique to negative observations, *Neg*_*unique*_, and the frequent patterns that are common on both cases, *PN*_*common*_. As outlined in Section 5, downstream ML models can leverage these patterns as input.

### 4.1. Benefits of Dichotomic Pattern Mining

**Figure 1:**
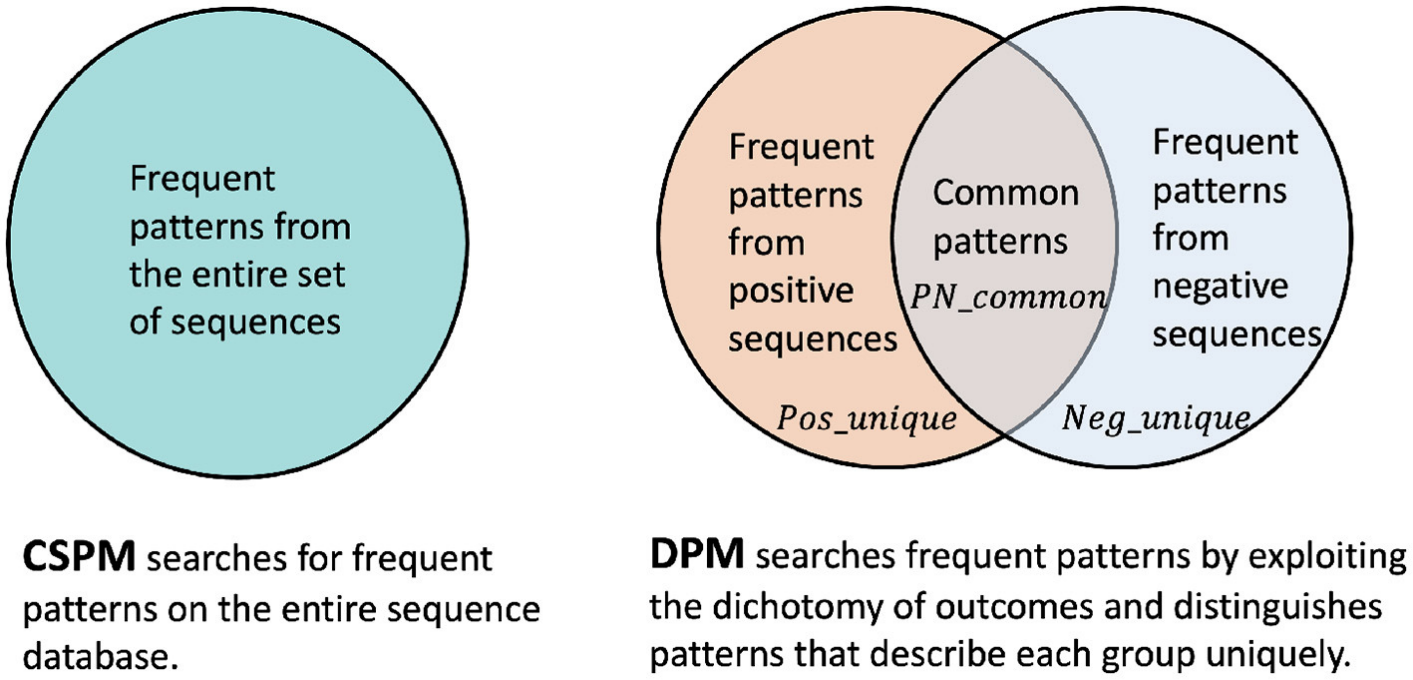
Traditional CSPM that operates on the entire sequence database vs. our DPM approach that exploits the dichotomy of outcomes to distinguish unique positive and negative patterns.

The traditional approach to identify recurring patterns consists of applying (C)SPM on the entire sequence database including both positive and negative sequences. Figure 1 illustrates a comparison between different pattern mining approaches as in traditional CSPM vs. our proposal for DPM.

The traditional CSPM serves as a baseline approach while DPM offers several benefits when compared to its traditional counterpart. First of all, in many practical scenarios, the classes of sequences are highly imbalanced. As such, setting a single static minimum frequency threshold for pattern mining on the entire sequence database is misleading. More concretely, setting a higher frequency threshold leads to the exclusion of useful recurring patterns in the minor class, whereas a lower threshold keeps too many redundant and arbitrary patterns from the major class. In both cases, the quality of patterns will be negatively impacted.

Moreover, DPM is an expressive approach to pattern mining in which constraint models for each class label can be configured specifically. Thus it seeks patterns that represent each outcome more significantly. Finally, from a scalability perspective, the traditional approach must run on the entire dataset as a single batch. Contrarily, decomposing the dataset eases the mining tasks. In fact, the dichotomic CSPMs can be executed in parallel. Computational complexity of DPM is therefore dependent on the CSPM algorithm applied to the sequence database, which depends on the size of the database, the number of constraints, or the number of attributes (Pei et al., 2007; Hosseininasab et al., 2019). Overall, we expect DPM to be more efficient in pattern mining (since it is applied to smaller datasets thanks to dichotomy) and DPM patterns to be more effective (since it captures dichotomy uniquely) in downstream consumption.

## 5. DPM as an Integration Technology

For sequential pattern mining with constraints, our tool Seq2Pat is readily available for mining applications that deal with data encoded as sequences of symbols. For continuous sequences, such as time series, discretization can be performed (Lin et al., 2007; Fournier-Viger et al., 2017). Previous work presents successful applications using streaming data from MSNBC, an e-commerce website, and online news. These are considerably large benchmarks with 900 K sequences of length more than 29 K, containing up to 40 K items (Hosseininasab et al., 2019).

**Figure 2:**
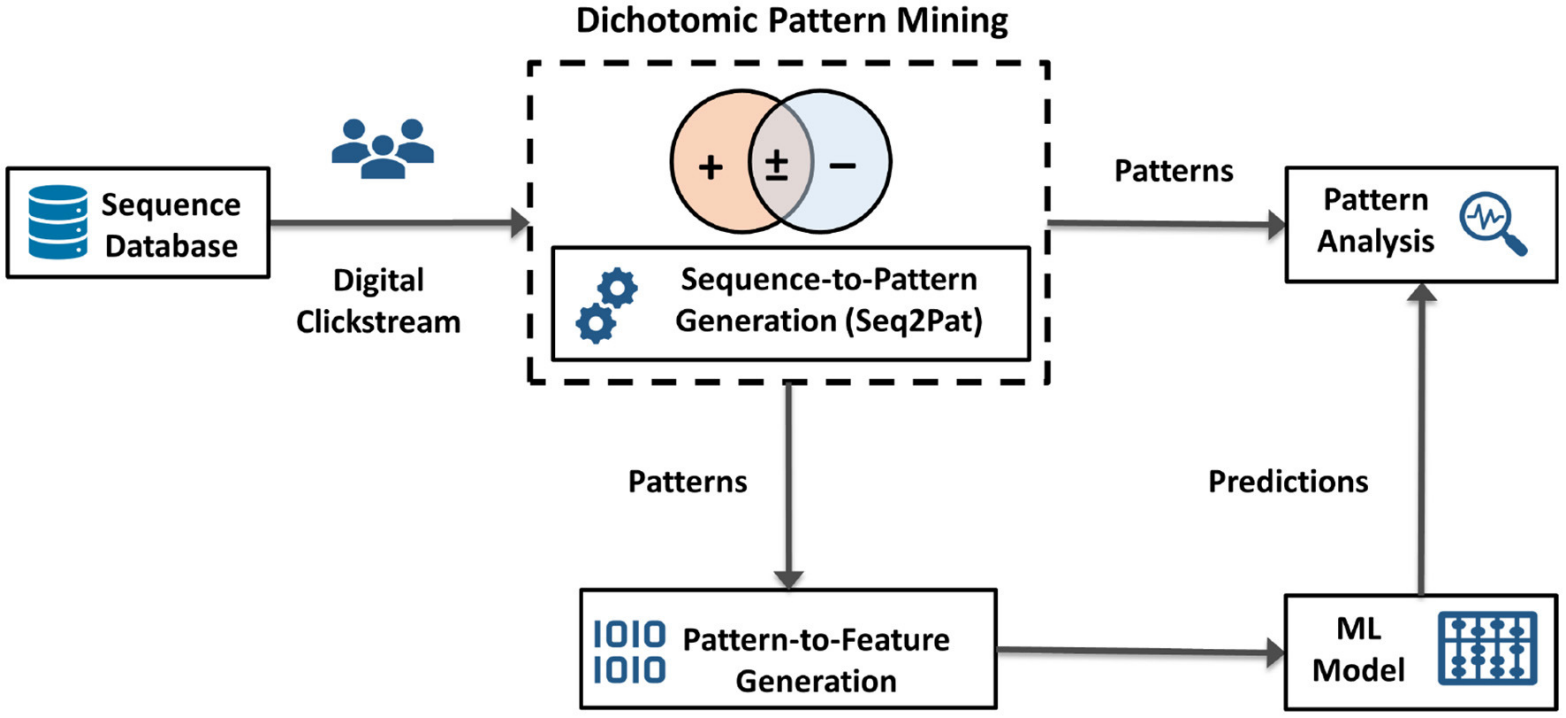
High-level system architecture for dichotomic pattern mining, embedded with sequence-to-pattern generation, as an integration technology between raw sequential data, e.g., clickstream, pattern analysis, pattern-to-feature generation, and machine learning models for downstream prediction tasks.

As illustrated in Figure 2, going beyond pattern mining, we envision DPM as an *integration technology* to enable other AI applications. DPM can be used to capture data characteristics for downstream AI models. DPM generates succinct representations from large volumes of data, e.g., digital clickstream activity. The patterns found then become consumable for subsequent machine learning models and pattern analysis. This generic process alleviates manual feature engineering and automates feature generation. Thus, our algorithm serves as an integration block between pattern mining algorithms and the subsequent learning task. In the next section, we present a demonstration of this integration for digital behavior analysis.

Note that the output of DPM as shown in Algorithm 1 is a set of frequent patterns, P, that provides insights into how the sequential behavior varies between populations. Using P, we learn new representations for sequences using sequence-to-pattern generation. Next, to create a feature vector for each sequence, we use pattern-to-feature generation to encode sequences. A typical featurization approach is one-hot encoding to indicate the existence of patterns. We further discuss these components on pattern and feature generation in our computation experiments. Overall, our end-to-end system architecture yields an automated feature extraction process that is generic and independent of the machine learning models applied to pattern embeddings. In the next section, we cover the case for digital behavior analysis whereby DPM acts as the integrator in real-world scenarios.

## 6. Digital Behavior Analysis

The application focus is on leveraging DPM to analyze digital behavior, and in particular, the analysis of large volumes of digital clickstream data. Clickstream data is ubiquitous, possess unique properties such as real-time and sequential ordering, and is challenging to work with as a streaming data source. This type of dataset is classified as *semi-structured data*. On the one hand, the clickstream data provides *unstructured text*, such as web pages. On the other hand, it yields sequential information where visits can be viewed as *structured event streams* representing customer journeys. Given the clickstream behavior of a set of users, we are interested in two specific aspects ranging from population-level to individual-level information extraction. At the population level, we are interested in finding the most frequent clickstream patterns across all users subject to a set of properties of interest. At the individual level, we are interested in downstream tasks such as intent prediction. Finally, an overarching theme over both levels is the interpretability of the results.

Our contribution to digital behavior analysis is to show that (i) constrained-based sequential pattern mining is effective in extracting knowledge from semi-structured data and (ii) DPM serves as an integration technology to enable downstream applications while retaining interpretability. The main idea behind our approach is first to capture the population characteristics and extract succinct representations from large volumes of digital clickstream activity using DPM. Then, the patterns found by DPM become consumable in machine learning models. Overall, our generic framework alleviates manual feature engineering, automates feature generation, and improves the performance of downstream tasks.

To demonstrate our approach, we explore two real-world clickstream datasets with positive and negative intents for product purchases based on shopping activity and intrusion behavior based on web visits. We apply our framework to find the most frequent patterns in digital activity and then leverage them in machine learning models for intent and intrusion prediction. Finally, we show how to extract high-level signals from patterns of interest.

## 7. Computational Experiments

In the following, we outline the goal of our experiments, describe the data (Section 7.1), the CSPM constraint models for sequence-to-pattern generation using Seq2Pat (Section 7.2), pattern-to-feature generation (Section 7.3), and downstream prediction models.

We present numeric results that compare the prediction accuracy on both customer intent prediction and intrusion datasets experimenting with simple to complex machine learning models. We also stress test the runtime behavior of pattern mining as a function of the number of patterns and constraints (Section 7.5.3). Finally, we study feature importance to drive insights and explanations from auto-generated features (Section 7.6).

To demonstrate the effectiveness of our DPM framework, we consider the following research questions:

Q1: How does DPM perform when compared to a standalone CSPM that does not exploit the dichotomy in outcomes?
Q2: How does DPM compare with state-of-the-art methods, such as LSTMs, that are specifically designed for sequential data?
Q3: How effective is DPM as an integration technology when the patterns it generates are used in prediction tasks?

To answer the above questions, we design two sets of experiments. In the first set of experiments, we compare the performance of DPM and the traditional CSPM applied to the entire dataset. In this controlled setup, the patterns found by each method is fed into a Logistic Regression (Cox, 1958) to make downstream predictions. We consider a simple modeling approach, such as Logistic Regression, to make the difference between the two approaches more pronounced. As mentioned in Section 4.1, due to class imbalance, variability in minimum frequency, and the distinction between constraint models, we expect DPM to be superior to CSPM in terms of prediction performance.

In the second set of experiments, we consider an array of machine learning models with different generalization capacities, including the state-of-the-art LSTM architecture from Requena et al. (2020). This allows us to quantify the best possible prediction performance achieved by DPM and position with respect to existing work. This also measures the effectiveness of our approach as an integration technology as well as an automated feature extractor from raw clickstream datasets. In these experiments, we expect DPM to be competitive, if not superior, to the previous literature.

### 7.1. Clickstream Datasets

We consider two digital clickstream datasets, namely the e-commerce shopper intent prediction dataset (Requena et al., 2020) for predicting the purchase intent at the end of a browsing session and intruder detection dataset (Kahn et al., 2016) for predicting whether the web session is made by an intruder.

#### 7.1.1. Shopper Intent Prediction Dataset

The dataset contains rich clickstream behavior on online users browsing a popular fashion e-commerce website (Requena et al., 2020). It consists of 203,084 shoppers' click sequences. There are 8,329 sequences with at least one purchase, while 194,755 sequences lead to no purchase. The sequences are composed of symbolized events as shown in Table 2 with length *L* between the range 5 ≤ *L* ≤ 155. Sequences leading to purchase are labeled as positive (+1); otherwise, labeled as negative (0), resulting in a binary intent classification problem.

**Table 2:** The symbols used to depict clickstream events in e-commerce shopper intent prediction dataset.

**Symbol**	**Event**
1	Page view
2	Detail (see product page)
3	Add (add product to cart)
4	Remove (remove product from cart)
5	Purchase
6	Click (click on result after search)

#### 7.1.2. Intruder Detection Dataset

The dataset (Kahn et al., 2016) consists of sequences of web pages visited by users. In addition to this, it contains labels denoting whether the web sessions were by genuine users or intruders. It has 253,561 click sequences of users out of which 250,098 instances have more than 1 click per sequence. 247,804 sequences of genuine users are labeled as positive (+1); the remaining 2,294 sequences are labeled as negative (0), resulting in a binary intrusion classification problem.

### 7.2. Sequence-To-Pattern Generation

The Step 2 in Algorithm 1 leaves the choice of the data mining approach to extract frequent patterns open. As mentioned earlier, in this step, we utilize the state-of-the-art Seq2Pat (Wang et al., 2022) to find sequential patterns that occur frequently. Seq2Pat takes advantage of the multi-valued decision diagram representation of sequences (Hosseininasab et al., 2019) and offers a declarative modeling language to support constraint reasoning. We developed Seq2Pat in a unique collaboration between academia and industry for large-scale mining operations to meet enterprise requirements and shared it with the community as an open-source library. The library is written in Cython to bring together the efficiency of a low-level C++ backend and the expressiveness of a high-level Python public interface (Behnel et al., 2011). Equipped with Seq2Pat, we next declare our constraint model, *C*_*type*_(·), to specify patterns of interest.

**Figure 3:**
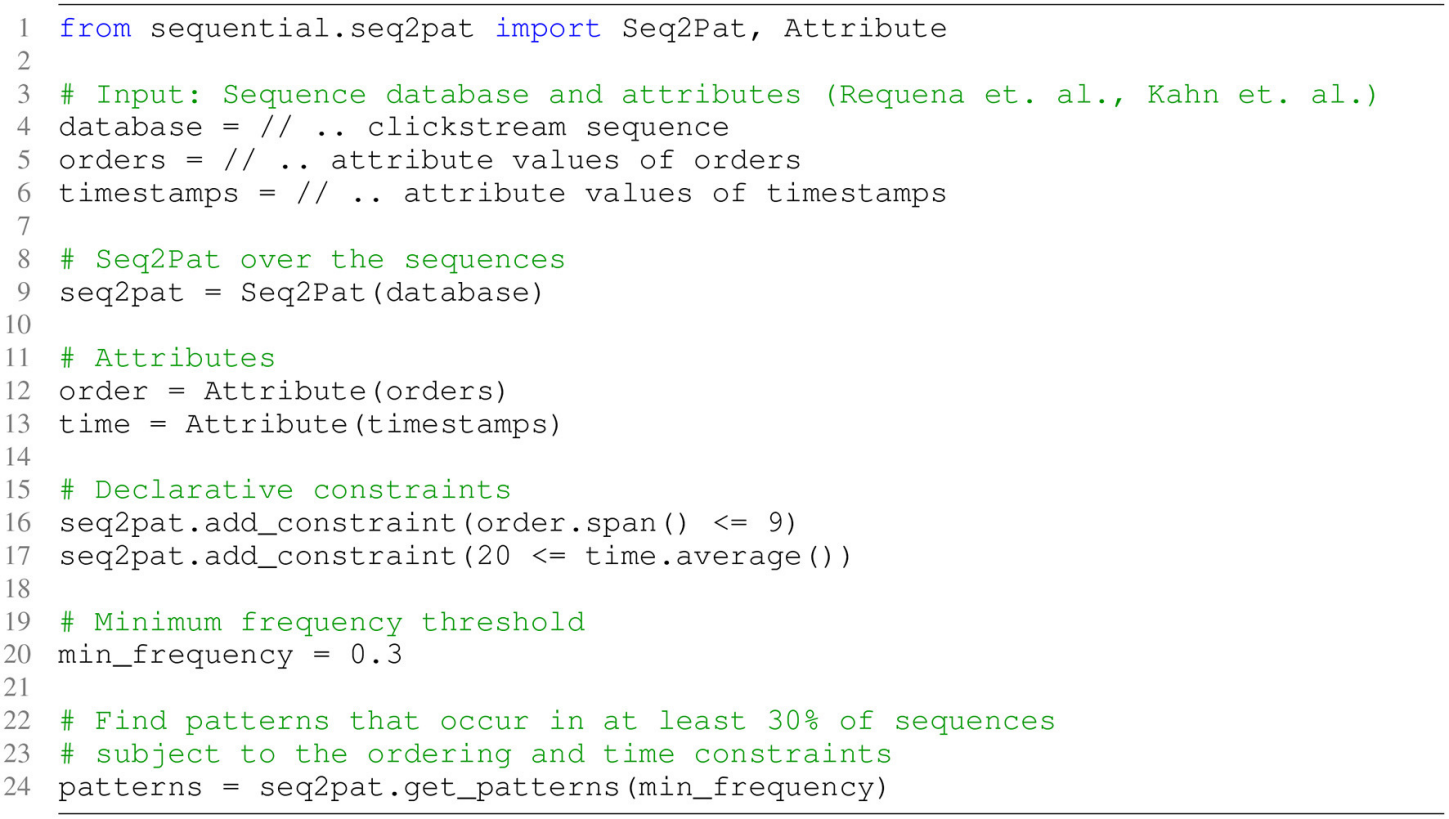
Seq2Pat model to enforce sequential pattern mining with constraints on the clickstream dataset in fashion e-commerce.

Figure 3 present the CSPM constraint model using the exact Seq2Pat implementation on the intent prediction dataset. The clickstream data serves as the sequence database (Line 3). In addition, we have two attributes for each event: the order in a sequence, $A_{order}$ (Line 5), and the dwell time on a page, $A_{time}$ (Line 6). Next, the Seq2Pat engine is created over the sequence database (Line 9). We then encapsulate this order and timestamp information in *Attribute* objects (Line 12–13) so that the user can interact with the raw data. The attribute object allows reasoning about the properties of the pattern. We enforce two constraints to seek interesting patterns. The first condition (Line 16) restricts the span of event order in a pattern to be ≤ 9. This ensures the maximum length of a pattern is 10. This condition is added to the system as a *constraint* together with the average constraint on time (Line 17). The average constraint seeks page views where customers spend at least 20 s. More precisely, we set *C*_*span*_($A_{order}$) ≤ 9 and *C*_*avg*_($A_{time}$)≥20_(*sec*)_. Finally, we set the minimum frequency threshold θ as the 30% of the total number of sequences for shopper intent prediction.

For the intruder detection dataset, the Seq2Pat constraint model for the intruder detection is almost identical, hence we omit the full implementation details. In this dataset, we seek for patterns of size ≤ 10, where the average time spent is at least 0.02 s, and the minimum frequency threshold is set to 0.1% for intruder detection.

Let us note that we find these values upon exploratory data analysis on the distribution of the datasets. Notice that these settings are not hyper-parameters to tune but rather characteristics of the datasets and a reflection of modeling preferences when seeking patterns of interest.

With the constraint model in Figure 3, Seq2Pat finds 457 frequent patterns in purchase sequences, *Pos*_*frequent*_, and 236 frequent patterns from the non-purchase sequences, *Neg*_*frequent*_, from the e-commerce shopper digital clickstreams. On intruder detection dataset, Seq2Pat finds 2006 frequent patterns in intruder sequences and 1006 frequent patterns in non-intruder sequences. There exists some overlap between *Pos*_*frequent*_ and *Neg*_*frequent*_ on both datasets.

### 7.3. Pattern-To-Feature Generation

Our next task is to convert frequent patterns into features that can be consumed by prediction models. As shown in the Venn diagram in Figure 1, DPM framework resorts to finding the unique patterns and the intersection from positive and negative outcomes.

In shopper intent prediction, when the sets of patterns from purchaser and non-purchaser are compared, we find 244 unique purchaser patterns, *Pos*_*unique*_, and 23 unique non-purchaser patterns, *Neg*_*unique*_. The groups share 213 patterns in common. In combination, we have 480 unique patterns *PN*_*union*_. To transform 480 unique patterns into a feature space, we consider a binary representation *via* one-hot encoding. For each sequence, we create a 480-dimensional feature vector with a binary indicator to denote the existence of a pattern.

For intruder detection, we follow the similar feature generation procedure, but find 1,894 unique intruder patterns, 894 unique non-intruder patterns, with 112 patterns being in common. In combination, we have 2,900 unique patterns to create the binary indicator in the second dataset.

### 7.4. [**Q1**] Comparison of DPM With CSPM

Let us start by quantifying the added value of DPM as opposed to applying CSPM on the entire dataset which serves as a baseline. Let us note that CSPM is a strong baseline. CSPM already incorporates sequential information and constraint-reasoning. As such, it is an advancement over classical pattern mining approaches, which we would like to improve further with DPM.

In this set of experiments, a Logistic Regression (LR) model is used for the shopper intent prediction and the intruder detection tasks. In setting this baseline, we consider a standard (logistic) regression model so that we can attribute the difference in the results to the choice of pattern mining approach. We first generate patterns from sequences, as explained in Section 7.2, and then generate features from patterns, as explained in Section 7.3. Then, the LR model is trained using either the extracted features by DPM or CSPM. The goal of this experiment is to demonstrate that DPM provides the necessary flexibility in the mining process, especially when sequences are largely imbalanced in classes. This is inherently the case in most practical scenarios such as we have many more visitors than purchasers in fashion e-commerce and only a fraction of intruders within regular access patterns. DPM helps to find patterns that are significantly presented in both major and minor classes, meanwhile it effectively excludes redundant patterns. Thus DPM is expected to be superior to standalone CSPM in supporting the downstream modeling.

#### 7.4.1. Prediction Model

We train a LR model on each of the two used datasets. We use 80% of the data as the train set and 20% as the test set and repeat this split 10 times for robustness. We compare the average results for each model based on Precision, Recall, F1 score, and the area under the ROC curve, aka AUC.

##### 7.4.1.1. Hyper-Parameter Tuning

On both datasets, we apply three-fold cross-validation for hyper-parameter tuning using the train set. We apply grid search on the regularization parameter C [0.001, 0.01, 0.1, 1, 10, 100, 1,000] for LR model. On shopper intent prediction dataset, a final parameter C is set to be 0.01 for the data using traditional CSPM features (referred to as LR_CSPM) and 0.1 for the data using DPM based features (referred to as LR_DPM) since these values provide the best performance in terms of AUC. On the intruder detection dataset, C has been set to be 100 and 0.1 for LR_CSPM and LR_DPM, respectively.

#### 7.4.2. Prediction Performance of DPM and CSPM Features With Logistic Regression

Table 3 summarizes the averaged performance and standard deviation of the LR model for LR_CSPM and LR_DPM. The best performance is marked in bold. We set the minimum frequency threshold to be 30% for shopper intent prediction and 0.1% for intruder detection. Notice that LR model is not able to utilize the sequential information of input by default. The auto-generated features by applying DPM or standalone CSPM enable this simple model to tackle the sequential knowledge. On both shopping and intrusion datasets, LR_DPM consistently outperforms LR_CSPM on *all metrics by a significant margin*. This highlights the effectiveness of DPM to exploit unique patterns in different classes.

**Table 3:** Comparison of averaged intent classification performance by using Logistic Regression model on CSPM and DPM features over 10 random Train-Test splits.

**Model**	**Precision(%)**	**Recall(%)**	**F1(%)**	**AUC(%)**
**Shopper intent prediction dataset**				
LR_CSPM	14.04 (±0.56)	36.25 (±4.59)	20.15 (±0.74)	78.67 (±0.38)
LR_DPM	**42.15** (±1.5)	**63.22** (±3.93)	**50.47** (±0.9)	**94.28** (±0.17)
**Intruder detection dataset**				
LR_CSPM	6.10 (±1.18)	13.90 (±5.04)	8.07 (±1.12)	76.98 (±0.85)
LR_DPM	**30.87** (±6.21)	**22.81** (±5.02)	**25.25** (±1.32)	**87.11** (±0.57)

For shopper intent prediction, DPM identifies 480 patterns while standalone CSPM finds only 236 patterns. Standalone CSPM is applied on the entire data and sequence classes are largely imbalanced with only 8,329 out of 203,084 sequences being positive. As a result, a single frequency threshold becomes too high to include the patterns that are frequent in positive sequences. Instead, most mined patterns are restricted to be the ones from negative sequences. Overall, patterns mined from the entire dataset does not transform into predictive features for intent classification. DPM overcomes such performance deterioration by exploiting the structure of the data.

Alternatively, we have the option to lower the minimum frequency to include more patterns, such as by setting the threshold in standalone CSPM to be 30% of positive sequences, i.e., 0.01% of the entire set of sequences. Then we obtain 4,615 patterns which bring redundant and arbitrary patterns from negative sequences since the threshold becomes too low. The performance is again in favor of DPM. For this dataset, we conclude that setting a single static frequency threshold, a high or low value, misguides the feature generation and adversely impacts the performance of the downstream prediction task.

For the intruder detection dataset, we obtain similar results as shown in Table 3, The number of patterns decreased from 2,900 by applying DPM to 988 by standalone CSPM with a minimum frequency threshold being 0.1%. DPM dominates CSPM performance across all metrics by a large margin again.

To conclude Q1, based on these experiments, we find that DPM is superior to standalone CSPM. Before proceeding with further experiments that combine DPM with more sophisticated models, let us examine the efficiency in terms of runtime in the next section.

#### 7.4.3. Runtime Performance

We report runtime performance of pattern mining on a machine with Linux RHEL7 OS, 16-core 2.2 GHz CPU, and 64 GB of RAM. We apply Seq2Pat to implement DPM and standalone CSPM. We impose the constraints as described in Section 7.2. For shopper intent prediction, the runtime of DPM is 117.49 s on positive sequences and 2088.34 s on negative sequences. Standalone CSPM runs for 2136.09 s. The runtime results are comparable since it is mostly dominated by the size of the majority class. For intruder detection, DPM runs for 0.2 s on positive sequences and 15.21 s on negative sequences while CSPM runs for 13.82 s. Overall, both DPM and CSPM achieve similar runtime and they both scale to sequence databases with 200,000+ and 250,000+ sequences. This is thanks to the underlying Seq2Pat library for an efficient implementation of constraint-based sequential pattern mining.

### 7.5. **[Q2 and Q3]** DPM as an Integration Technology and Comparison With the State-Of-The-Art in Prediction Performance

The goal of our next set of experiments is to quantify the effectiveness of DPM as an integration technology and an automated feature extractor. For that purpose, we consider an array of machine learning models that are more sophisticated than Logistic Regression and exhibit varying generalization capacity.

#### 7.5.1. Prediction Models

As shown in Table 4, we consider four different models with different strengths using CSPM or DPM features either in standalone or in combination with LSTM.

**Table 4:** The set of machine learning models considered and their feature space.

**Model**	**Features space**
LightGBM_{CSPM, DPM}	CSPM or DPM features
Shallow_NN_{CSPM, DPM}	CSPM or DPM features
LSTM	Clickstream Data
LSTM+{CSPM, DPM}	Clickstream data + CSPM or DPM patterns

1. LightGBM light gradient boosting machines (Ke et al., 2017).
2. Shallow_NN shallow neural network using one hidden layer.
3. LSTM The state-of-the-art long short-term memory network (Hochreiter and Schmidhuber, 1997) from Requena et al. (2020) that uses input sequences as-is. LSTM applies one hidden layer on the output of the last layer followed by a fully connected layer to make intent prediction.
4. LSTM+{CSPM,DPM} The LSTM model boosted with pattern embeddings from CSPM or DPM. The model uses the same architecture with LSTM, with the only difference being that pattern based features are concatenated to the output of LSTM and are used together as input of the hidden layer.

Table 4 also shows the different feature spaces used by the compared models. Note models such as LightGBM and Shallow_NN cannot operate on semi-structured clickstream data since they cannot accommodate recurrent sequential relationships. Contrarily, more sophisticated architectures, such as LSTM can work directly with the input. For the former, our approach allows these relatively simpler models to work with sequence data. For the latter, our approach augments advanced models by incorporating pattern embeddings into the feature space. We use the same train-test split as described in the first set of experiments and repeat it 10 times for robustness.

##### 7.5.1.1. Hyper-Parameter Tuning

Similar to the parameter tuning in previous experiment, we apply three-fold cross-validation for hyper-parameter tuning using the train set. We apply grid search on the number of iterations [400, 600, 800, 1,000] for LightGBM, number of nodes in the hidden layer [32, 64, 128, 256, 512] for Shallow_NN and LSTM models, number of LSTM units [16, 32, 64, 128]. We use 10% of train set as a validation set to determine if training meets early stop condition. When the loss on validation set stops decreasing steadily, training is terminated. The validation set is used to determine a decision boundary on the predictions for the highest F1 score. The final parameters for shopper intent prediction models are 400 iterations for LightGBM, 64 nodes for shallow_NN, 32 LSTM units in LSTM models with 64 and 128 nodes in hidden layers. Intruder detection has slightly different parameters where LightGBM still gets 400 iterations, but the tuning results have 32 nodes for shallow_NN, 16 and 32 LSTM units in LSTM and LSTM+{CSPM, DPM} model, respectively, with each having 32 nodes in hidden layers.

#### 7.5.2. Prediction Performance With Sophisticated Models

Table 5 presents the average results that compare the performance of the models on the two datasets. The best performance is marked in bold. For feature space, we either use the patterns found by CSPM or DPM, the original clickstream events in the raw data, or their combination. For shopper intent prediction, using auto-generated DPM features, LightGBM, and Shallow_NN models achieve a performance that closely match the results given in the reference work (Requena et al., 2020). The difference is, models in Requena et al. (2020) use hand-crafted features, while we automate the feature generation process here. Notice that without using clickstream events, the performance of both two models using DPM features significantly outperform those using CSPM features. When a more sophisticated model such as LSTM is used, it outperforms LightGBM and Shallow_NN. When the LSTM model is combined with CSPM or DPM, LSTM+CSPM achieves the best Precision among compared models while it achieves a similar performance with LSTM in terms of other metrics. Compared to LSTM and LSTM+CSPM, LSTM+DPM yield a substantial increase in Recall, and consequently, the highest F1 score. LSTM+DPM is also superior to others in terms of AUC.

**Table 5:** Comparison of averaged prediction performance by different methods over 10 random Train-Test splits.

**Model**	**Precision(%)**	**Recall(%)**	**F1(%)**	**AUC(%)**
**Shopper intent prediction dataset**				
LightGBM_CSPM	25.11 (±2.25)	33.34 (±3.52)	28.42 (±0.59)	83.81 (±0.43)
LightGBM_DPM	44.70 (±1.92)	63.15 (±4.65)	52.20 (±0.65)	94.98 (±0.15)
Shallow_NN_CSPM	22.62 (±2.06)	37.78 (±3.95)	27.18 (±0.59)	83.17 (±0.45)
Shallow_NN_DPM	44.40 (±2.18)	64.11 (±4.57)	52.31 (±0.54)	95.00 (±0.17)
LSTM	54.96 (± 1.77)	69.53 (±4.31)	61.28 (±0.95)	96.41 (±0.15)
LSTM+CSPM	**55.29** (±1.78)	69.10 (±3.00)	61.36 (±0.83)	96.42 (±0.15)
LSTM+DPM	54.35 (±2.40)	**73.64** (±4.70)	**62.39** (±0.81)	**96.76** (±0.12)
**Intruder detection dataset**				
LightGBM_CSPM	7.27 (±1.27)	12.66 (±4.35)	8.94 (±1.39)	77.25 (±0.90)
LightGBM_DPM	32.50 (±3.52)	23.31 (±3.84)	26.81 (±2.47)	86.35 (±0.72)
Shallow_NN_CSPM	6.51 (±1.16)	13.34 (±4.14)	8.50 (±1.21)	77.30 (±0.87)
Shallow_NN_DPM	39.55 (±6.15)	22.28 (±4.14)	27.94 (±2.46)	85.28 (±0.81)
LSTM	42.62 (±6.19)	39.24 (±6.20)	40.31(±0.98)	95.20 (±0.36)
LSTM+CSPM	42.20 (±5.49)	**41.52** (±3.64)	**41.46** (±1.64)	**95.42** (±0.47)
LSTM+DPM	**50.81** (±8.24)	35.89 (±3.63)	**41.46** (±1.59)	95.14 (±0.26)

On the intruder detection dataset, again automatic feature extraction provides LightGBM and Shallow_NN an opportunity to tackle sequential input. As before, LSTM model using clickstream sequences significantly improves the prediction performance compared to LightGBM and Shallow_NN. Further, LSTM+CSPM performs better at Recall, Precision and slightly improves at AUC. Overall, LSTM+DPM achieves the best precision performance and the same highest F1 score as LSTM+CSPM.

To conclude Q2 and Q3, based on the results observed in two different datasets across four modeling choices, we conclude that the features extracted automatically *via* DPM boost ML models in the downstream task for intent and intrusion prediction, especially for the models such as LightGBM and Shallow_NN that lack the capability to operate on sequential clickstream data. Using DPM achieves significantly better performance than using CSPM for these two models. As for the model that can deal with sequence data such as LSTM, the improvement by using DPM over CSPM or only clickstream sequence becomes leveled off due to the sophistication of the model itself, which is as shown in the intruder detection task, while we observe a clear boost by using DPM features in the task for intent prediction.

#### 7.5.3. Runtime Performance

Let us complement the model performance results with observations in the runtime as a function of different constraint models. We present the results for the fashion e-commerce dataset only since the findings are similar. We apply Seq2Pat on the positive set with 8,329 clickstream sequences. We impose the same types of constraint as described in Section 7.2 while we vary the constraint on the minimum average time spent on pages. To stress-test the runtime, we set the minimum frequency θ = 2 which returns almost all the feasible patterns.

**Figure 4:**
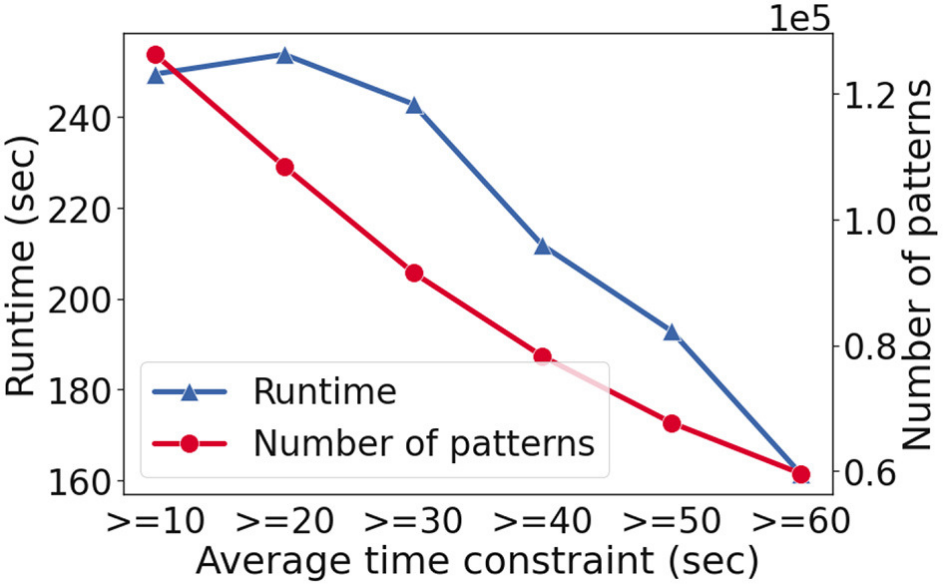
The runtime (y-axis-left) and the number of patterns found (y-axis-right) with varying constraints (x-axis) on the fashion e-commerce dataset. In this setting, the minimum frequency threshold, θ, set to 2, to stress test runtime performance.

Figure 4 shows the runtime in seconds (y-axis-left) and the number of patterns found (y-axis-right) as the average constraint increases (x-axis). As the constraint becomes harder to satisfy, the number of patterns goes down as expected. The runtime for the hardest case is ~250 s while we observe speed-up as constraint reasoning becomes more effective.

### 7.6. Feature Importance

Finally, we study feature importance to drive high-level insights and explanations from auto-generated DPM features. We examine the Shapley value (Lundberg et al., 2020) of features from the LightGBM model in the fashion e-commerce dataset.

**Figure 5:**
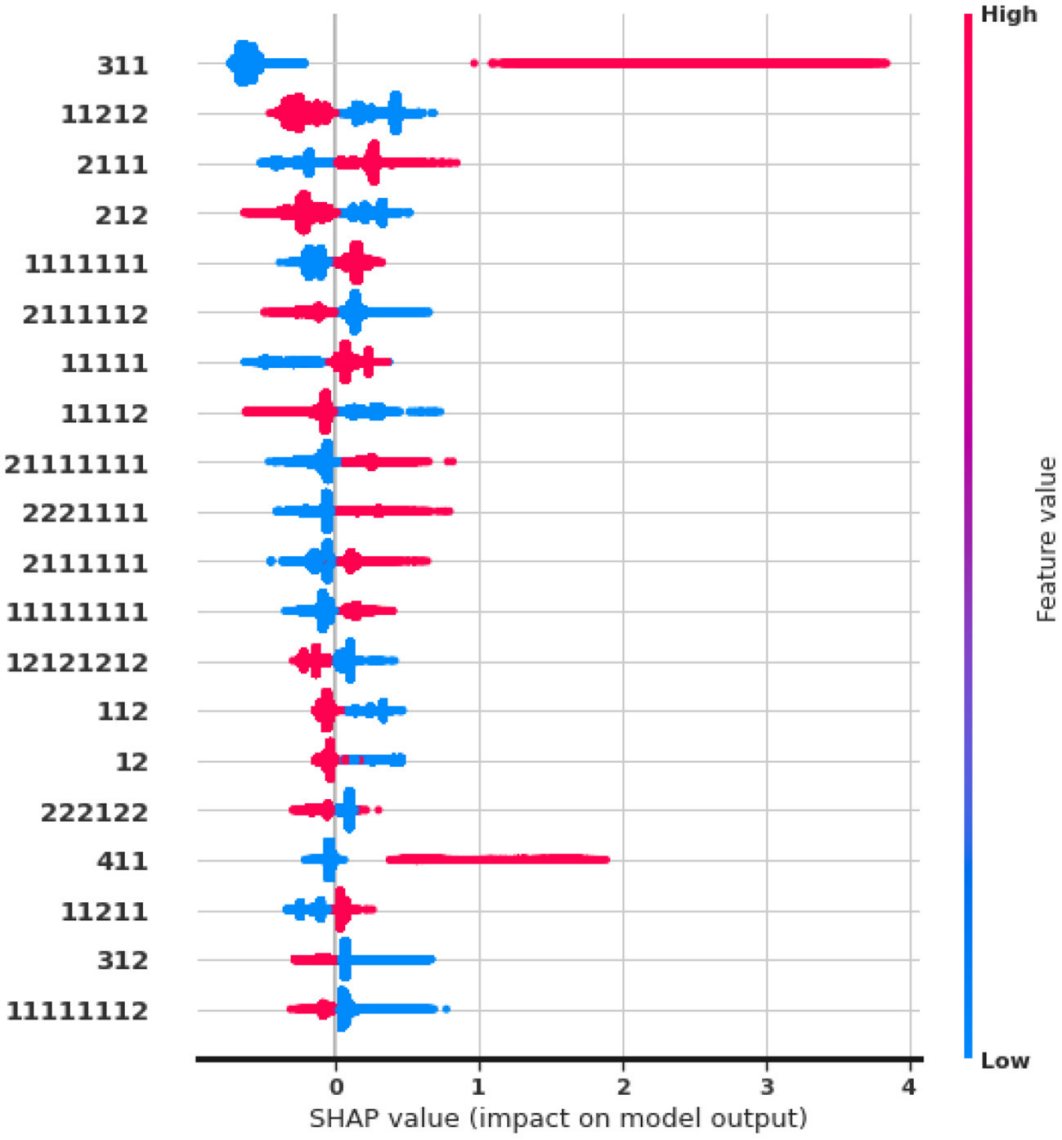
SHAP values of auto-generated Seq2Pat features. Top-20 features ranked in descending importance. Color indicates high (in red) or low (in blue) feature value. Horizontal location indicates the correlation of the feature value to a high or low model prediction.

Figure 5 shows the top-20 features with the highest impact. Our observations match previous findings in Requena et al. (2020). The pattern 〈3, 1, 1〉 provides the most predictive information, given that the symbol (3) stands for adding a product. Let us remind that Table 2 describes the meaning of symbols. Repeated page views as in 〈1, 1, 1, 1, 1, 1, 1〉, or specific product views, 〈2, 1, 1, 1〉 are indicative of purchase intent, whereas web exploration visiting many products, 〈1, 1, 2, 1, 2〉, are more negatively correlated to a purchase. Interestingly, searching actions 〈6〉 have minimum impact on buying, raising questions about the quality of the search and ranking systems. Our frequent patterns also yield new insights not covered in the existing hand-crafted analysis. Most notably, we discover that removing a product but then remaining in the session for more views, 〈4, 1, 1〉 is an important feature, positively correlated with a purchase. This scenario, where customers have specific product needs, hints at the missed business opportunity to create incentives such as prompting virtual chat or personalized promotions.

## 8. Conclusion

Pattern mining is an essential part of data analytics and knowledge discovery from sequential databases. It is a powerful tool, especially when combined with constraint reasoning to specify desired properties. In this paper, we presented a simple procedure for Dichotomic Pattern Mining that operates over semi-structured clickstream datasets. The approach learns new representations of pattern embeddings. This representation enables simple models, which cannot handle sequential data by default, to predict from sequences. Moreover, it boosts the performance of more complex models with feature augmentation. Experiments on digital behavior analysis demonstrate that our approach is an effective integrator between automated feature generation and downstream tasks. Finally, as shown in our feature importance analysis, the representations we learn from pattern embeddings remain interpretable.

We have only considered the dichotomy between binary classes. Extending Dichtomic Pattern Mining to effectively deal with multi-class classification problems is a natural next direction. Additionally, sequence databases reach large-scales quickly. It remains open whether it is possible to design a *distributed* version of CSPM and DPM algorithms so that we can take advantage of modern architectures, including GPUs, to scale beyond datasets considered in our experiments.

## Data Availability Statement

The dataset used in our experiments are public benchmarks that are available from the references therein and are commonly used in this line of research. Shopper Intent Prediction Dataset: https://github.com/coveooss/shopper-intent-prediction-nature-2020. Intrusion Detection Dataset: https://www.kaggle.com/danielkurniadi/catch-me-if-you-can.

## Author Contributions

All authors contributed equally to this study and the write-up of the manuscript. All authors read and approved the final manuscript.

## Conflict of Interest

SG, SY, XW, BC, and SK were employed by the company Fidelity Investments, United States.

## Publisher's Note

All claims expressed in this article are solely those of the authors and do not necessarily represent those of their affiliated organizations, or those of the publisher, the editors and the reviewers. Any product that may be evaluated in this article, or claim that may be made by its manufacturer, is not guaranteed or endorsed by the publisher.

## Footnotes

^1^ https://github.com/fidelity/seq2pat

^2^ https://github.com/fidelity/seq2pat/tree/master/notebooks/dichotomic_pattern_mining.ipynb
